# Sex and gender disparity in pathology, disability, referral pattern, and wait time for surgery in workers with shoulder injury

**DOI:** 10.1186/s12891-016-1257-7

**Published:** 2016-09-21

**Authors:** Helen Razmjou, Sandra Lincoln, Iona Macritchie, Robin R. Richards, Danielle Medeiros, Amr Elmaraghy

**Affiliations:** 1 Holland Orthopaedic & Arthritic Centre, Sunnybrook Health Sciences Centre, Toronto, Canada; 2 Department of Physical Therapy, Faculty of Medicine, University of Toronto, Toronto, Canada; 3 Sunnybrook Research Institute, Sunnybrook Health Sciences Centre, Toronto, Canada; 4 Toronto Rehabilitation Institute, Lyndhurst Centre, Brain & Spinal Cord Rehabilitation Program, Toronto, Canada; 5 Division of Orthopedic Surgery, Department of Surgery, Sunnybrook Health Sciences Centre, Toronto, Canada; 6 Department of Orthopedic Surgery, Faculty of Medicine, University of Toronto, Toronto, Canada; 7 Department of Orthopaedic Surgery, St. Joseph’s Health Centre, Toronto, Canada

**Keywords:** Sex, Gender disparity, Injured workers, Shoulder

## Abstract

**Background:**

The role of sex as an important biological determinant of vulnerability to sustaining injury and gender as a social determinate of access to resources, referral for medical care and perceived disability remains conflicted in injured workers. The purpose of this study was to examine sex and gender disparity following a compensable work-related shoulder injury.

**Methods:**

This study involved cross-sectional analyses of data of two independent samples of workers with shoulder injury. Measures of disability and pain were the Quick Disabilities of the Arm, Shoulder and Hand (QuickDASH) and Numerical Pain Rating Scale (NPRS) for patients seen at an Early Shoulder Physician Assessment (ESPA) program and the American Shoulder and Elbow Surgeons (ASES) assessment form and Visual Analogue Scale (VAS) for the sample who underwent surgery.

**Results:**

The files of 1000 (443 females, 557 men) consecutive patients seen at an ESPA program and 150 (44 females, and 106 men) consecutive patients who underwent rotator cuff surgery (repair or decompression) were reviewed. Significant gender disparity was observed in the referral pattern of injured workers seen at the ESPA program who were referred for surgical consultation (22 vs. 78 % for females and males respectively, *p <* 0.0001). The independent rotator cuff surgical group had a similar gender discrepancy (29 % vs. 71 %, *p <* 0.0001). The timeframe from injury to surgery was longer in women in the surgical group (*p =* 0.01). As well, women waited longer from the date of consent to date of surgery (*p =* 0.04). Women had higher incidence of repetitive injuries (*p =* 0.01) with men reporting higher incidence of falls (*p =* 0.01). Women seen at the ESPA program were more disabled than men (*p =* 0.02). Women in both samples had a higher rate of medication consumption than men (*p =* 0.01 to <0.0001). Men seen at the ESPA program had a higher prevalence of full thickness rotator cuff tears (*p <* 0.0001) and labral pathology (*p =* 0.01). However, these pathologies did not explain gender disparity in the subsample of ESPA who were referred for surgical consultation or those who had surgery.

**Conclusions:**

Sex and gender disparity exists in workers with shoulder injuries and is evident in the mechanism of injury, perceived disability, medication consumption, referral pattern, and wait time for surgery.

## Background

Rotator cuff injuries are known to be the most common source of disability of the work related shoulder conditions [1, 2] imposing a burden on health care and workers compensation systems [3–5]. Recent systematic reviews have shown that injured workers with shoulder conditions consistently report poorer outcomes than patients without work-related injuries [6, 7] and compensated shoulder injuries are associated with higher levels of dissatisfaction and disability with longer time frames to return to full activities or gainful employment following surgery [8–15].

As noted, the negative impact of compensated shoulder injuries is a well-known fact. However, role of “sex” as a biological predictor of sustaining an injury or developing pathology and “gender” as a psychosocial determinant of access to resources, being referred for medical care or perceived disability in the injured workers have not been well examined.

Biological differences in anatomy, hormones, aerobic capacity and strength [16–19] may increase women’s vulnerability in the workplace environment and increase the risk of sustaining certain pathologies. As well, biological differences in neuromuscular control system are reported to contribute to a higher rate of musculoskeletal disorders in women [20]. Gender-related differences in psychological and economic factors [20, 21] and clinicians’ bias toward prioritizing male referrals for specialized medical consultation [22–24] may also affect the quality of care provided to women.

Most of the information on workers with shoulder injuries comes from studies with a small sample of injured workers within a larger group of patients. In one study that has examined the association between gender and an active compensated shoulder claim, the subsample of injured women reported less satisfaction with surgery than women without a work-related injury [25]. In other studies, women reported more post-operative pain than their male counterparts [12] and reported higher inability to perform certain shoulder tasks [11]. However, considering that these studies used a subsample of injured workers, these findings may not necessarily be applicable to the injured worker population when studied in isolation. Using samples that have both compensable and non-compensable injuries may confound the analysis of sex and gender disparity due to the complex interplay of biological, cultural and political factors that exist in the factor of disability and the binominal factor of man/women. Sex and gender disparity in injured workers would be more accurately explored if the confounding factor of a compensable injury is accounted for by including only injured workers.

The purposes of this study were to examine 1) the observed differences between men and women in age, mechanism of injury, pathology, perceived disability and medication consumption which were affected by both biological and non-biological factors and 2) gender disparity in the referral pattern for recommending surgery and wait time to have surgery following a compensable shoulder injury.

## Methods

### Design

This study involved cross-sectional data analyses of two independent samples of workers with an active compensable shoulder injury who were referred for either an expedited assessment or surgery.

### Patient population

The first sample comprised of consecutive injured workers seen at an Early Shoulder Physician Assessment (ESPA) program, whose data were examined retrospectively (ESPA group). They were workers with shoulder injuries who had not progressed in their recovery or return to work (RTW) plan within 16 weeks of the injury or reoccurrence and had a variety of diagnoses including impingement syndrome, rotator cuff tendinitis, partial or full thickness rotator cuff tear, adhesive capsulitis, or labral pathologies. Patients were seen by an orthopedic surgeon and a physical therapist. Recommendations for further treatment such as conservative or surgical management were documented. Data of the subsample of ESPA group who were referred for surgical consultation were examined separately. Approval for using the retrospective data of this sample was obtained from the Research Ethics Board of the Sunnybrook Health Sciences Centre.

The second sample involved an independent sample of consecutive injured workers who had an expedited rotator cuff related surgery and had participated in a prospective study (surgical group). The inclusion criteria for this sample included an active work-related shoulder injury, age ≥18 years, diagnosis of tendonitis, partial or full-thickness rotator cuff tear confirmed by MRI or US. Patients with evidence of advanced osteoarthritis of the glenohumeral joint, inflammatory arthropathy, concurrent pathology of Superior Labral Anterior and Posterior (SLAP) lesions or Bankart lesions that required a repair were excluded. Arthroscopic rotator cuff decompression (acromioplasty, lateral resection of clavicle) was performed for osseous impingement, acromioclavicular arthritis or partial thickness rotator cuff tears where arthroscopic repair was conducted for full thickness rotator cuff tears. Low grade partial tears of biceps (<50 %) were debrided. Biceps tenodesis or tenotomy was conducted for high-grade tears of the tendon as appropriate. The size of rotator cuff tear (largest dimension) was categorized as small < 1 cm, moderate (1–3 cm.), large (>3–5 cm.), and massive (>5 cm.) [26]. All patients in the surgical group had provided consent to participate in research and the study was approved by the Research Ethics Board of the Sunnybrook Health Sciences Centre.

### Outcome measures

Patient-related outcome measures which were completed at the time of assessment in the ESPA group included the numeric pain rating scale (NPRS), and the Quick Disabilities of the Arm, Shoulder and Hand (QuickDASH) [27].

Outcome measures in the surgical group included the Visual Analogue Scale (VAS) [28] and the American Shoulder and Elbow Surgeons (ASES) assessment form [29].

The NPRS and VAS use a 0 to 10 scale with 0 being no pain and 10 being the worst imaginable pain and are valid for clinical use [30, 31]. Both the QuickDASH and ASES measures have established validity and reliability in patients with shoulder complaints [27, 32–34] and have shown high correlation with one another [25, 35].

### Referral pattern and wait time

The referral pattern was examined by exploring differences in the proportion of men and women in each sample. Wait time reflected time between date of injury and date of assessment in the ESPA group and those who were referred for surgical consultation. In the independent surgical group, wait time reflected time between date of injury and date of surgery. In addition, the time period between the date patient consented to surgery and date of actual surgery was examined in the surgical group.

### Statistical analysis

Descriptive statistics were conducted in each sample to examine potential sex and gender differences in demographics (e.g. age, medication use, mechanism of injury, type of pathology/surgery), pain (NPRS and VAS) and disability (as measured by QuickDASH or ASES). The Chi square (*χ*^2^) tests examined proportions between men and women. To examine the goodness of fit in 2 × 2 contingency tables and magnitude of meaningful difference between the number of men and women in each group, phi coefficients were calculated and Cohen’s effect size criteria [36] were used to interpret the effect sizes (0.1 = small, 0.3 = medium and 0.5 = large). Categorical data were examined by Chi square and Fisher’s Exact tests as appropriate and continuous data were examined by independent student t-tests or Wilcoxon two sample test depending on normality of data.

## Results

### ESPA group

Files of 1000 consecutive patients were reviewed retrospectively. The sample included 443 females (44 %) and 557 men (56 %) with the mean age of 49 (11), range 18–77 years (*χ*^2^ 12.99, *p =* 0.0003, Phi coefficient = 0.11, 95 % CI = 0.07–0.16, Effect size = small).

### ESPA subgroup

Of 1000 patients seen at the ESPA, 169 (17 %) patients were referred for surgical consultation after the first assessment. This subsample included 38/169 (22 %) women and 131/169 (78 %) men. There was a statistically significant difference in the proportion of men and women referred for surgical consultation (*χ*^2^ = 51.17, *p <* 0.0001, Phi coefficient = 0.55, 95 % CI = 0.46–0.64, Effect size = large).

### Surgical group with rotator cuff pathology

Pre-operative data of 150 consecutive patients who had participated in an independent prospective study were reviewed. This sample included 44 females (29 %) and 106 men (71 %), mean age 52, range 27–75 years (*χ*^2^ = 25.63, *p <* 0.0001, Phi coefficient = 0.41, 95 % CI = 0.31–0.51, Effect size = medium).

### Sex/gender related differences

Tables 1, 2 and 3 show the characteristics of the groups. Females’ age was comparable to males in all three samples.

**Table 1:** Sex/Gender differences in the ESPA group (*N =* 1000)

Variables (Mean, SD)/(N/%)	Women (%)	Men (%)	Statistics *P* values
Age	49 (11)	49 (11)	ttest = 0.65, *p =* 0.51
Referral pattern	443 (44 %)	557 (56 %)	*χ* ^2^ = 12.99, *p =* 0.0003 Phi coefficient = 0.11 95 % CI = 0.07-0.16 Effect size = Small
Wait time to assessment (months)	2.68 (0.98)	2.63 (0.80)	ttest = 0.84, *p =* 0.40
Affected Side			
• Right • Left • Bilateral	269 (61 %) 162 (37 %) 12 (3 %)	321 (58 %) 225 (40 %) 11 (2 %)	*χ* ^2^ = 1.9, *p =* 0.38
Mechanism of injury			
• Repetitive activities • Fall • Traumatic • Push/pull	69 (16 %) 54 (12 %) 47 (11 %) 175 (40 %)	58 (10 %) 100 (18 %) 53 (10 %) 237 (43 %)	*χ* ^2^ = 5.93, *p =* 0.01 *χ* ^2^ = 6.29, *p =* 0.01 *χ* ^2^ = 0.32, *p =* 0.57 *χ* ^2^ = 0.94, *p =* 0.33
Medication use			
• Non-narcotic analgesics • Anti-inflammatory • Muscle relaxants	213 (48 %) 258 (58 %) 42 (9 %)	229 (41 %) 237 (42 %) 19 (3 %)	*χ* ^2^ = 4.85, *p =* 0.03 *χ* ^2^ = 24.29, *p <* 0.0001 *χ* ^2^ = 15.87, *p <* 0.0001
Type of pathology			
• FTRCT • PTRCT • Impingement syndrome • Biceps pathology • Adhesive capsulitis • Labral pathology • Instability	28 (6 %) 95 (21 %) 231 (52 %) 172 (39 %) 35 (8 %) 0 (0 %) 8 (2 %)	93 (17 %) 128 (23 %) 289 (52 %) 220 (39 %) 43 (8 %) 10 (2 %) 14 (3 %)	*χ* ^2^ = 24.97, *p <* 0.0001 RR = 0.38^a^, 0.25–0.57 *χ* ^2^ = 0.34, *p =* 0.56 *χ* ^2^ = 0.006, *p =* 0.93 *χ* ^2^ = 0.05, *p =* 0.82 *χ* ^2^ = 0.02, *p =* 0.91 FET = 0.01, *p =* 0.01 *χ* ^2^ = 0.57, *p =* 0.45

FET Fisher's Exact Test, *χ* ^2^ = Chi square

^a^A risk ratio of 0.38 is expressed as women having decreased risk of presenting with FTRCT by 62 %: 100 × (1–0.38) %

**Table 2:** Sex/Gender differences in the ESPA subgroup referred for surgical consultation (*N =* 169)

Variables (Mean, SD)/(N/%)	Women (%)	Men (%)	Statistics *P* values
Age	54 (10)	53 (10)	ttest = 0.58, *p =* 0.56
Referral pattern	38 (22 %)	131 (78 %)	*χ* ^2^ = 51.17, *p <* 0.0001 Phi coefficient = 0.55 95 % CI = 0.46–0.64 Effect size = Large
Wait time to assessment (months)	2.83 (0.98)	2.46 (0.80)	ttest = 1.04, *p =* 0.30
Affected Side			
• Right • Left	21 17	84 47	*χ* ^2^ = 0.98, *p =* 0.32
Mechanism of injury			
• Repetitive activities • Fall • Traumatic • Push/pull	5 (13 %) 54 (12 %) 3 (8 %) 13 (34 %)	7 (5 %) 100 (18 %) 16 (12 %) 50 (38 %)	*χ* ^2^ = 2.70, *p =* 0.10 *χ* ^2^ = 0.11, *p =* 0.73 *χ* ^2^ = 0.55, *p =* 0.46 *χ* ^2^ = 0.20, *p =* 0.66
Medication use			
• Non-narcotic analgesics • Anti-inflammatory • Muscle relaxants	20 (53 %) (58 %) 0 (0 %)	57(44 %) 237(42 %) 3(2 %)	*χ* ^2^ = 0.98, *p =* 0.32 *χ* ^2^ = 0.01, *p =* 0.92 FET = 0.46, *p =* 1.80
Type of pathology			
• FTRCT • PTRCT • Impingement syndrome • Biceps pathology • Labral pathology • Instability	15 (39 %) 13 (34 %) 9 (24 %) 14 (37 %) 0 (0 %) 3 (2 %)	66 (50 %) 30 (23 %) 19 (15 %) 49 (37 %) 4 (40 %) 5 (3 %)	*χ* ^2^ = 1.40, *p =* 0.24 *χ* ^2^ = 1.98, *p =* 0.16 *χ* ^2^ = 1.79, *p =* 0.18 *χ* ^2^ = 0.004, *p =* 0.94 FET = 1.18, *p =* 0.28 FET = 0.18, *p =* 0.38

FET Fisher's Exact Test

*χ* ^2^ = Chi square

**Table 3:** Sex/Gender differences in the independent rotator cuff surgical group

Variables (Mean, SD)/(N/%)	Women (%)	Men (%)	Statistics *P* values
Age	51 (10)	52 (8)	ttest = 0.43, *p =* 0.66
Referral pattern (N,%)	44 (29 %)	106 (71 %)	*χ* ^2^ = 25.63, *p <* 0.0001 Phi coefficient = 0.41, 95 % CI = 0.31–0.51 Effect size = Medium
Wait time			
Wait time1 wait time2	20 (13) 95 (50)	14 (12) 77 (41)	Wilcoxon test = 2.61, *p =* 0.01 Wilcoxon test = 2.08, *p =* 0.04
Affected Side			
• Right • Left • Bilateral	27 (61 %) 14 (32 %) 3 (7 %)	51 (48 %) 44 (42 %) 11 (10 %)	FET = 0.01, *p =* 0.36
Side operated on			
• Right • Left	57 (73 %) 21 (27 %)	48 (59 %) 34 (41 %)	*χ* ^2^ = 1.36, *p =* 0.24
Mechanism of injury			
• Insidious • Repetitive activities • Fall • Traumatic • Other	4 (9 %) 10 (23 %) 11 (25 %) 16 (36 %)	4 (4 %) 13 (12 %) 41 (39 %) 37 (35 %)	FET = 0.13, *p =* 0.23 *χ* ^2^: 2.62, *p =* 0.11 *χ* ^2^: 2.57, *p =* 0.11 *χ* ^2^ = 0.03:, *p =* 0.86
Pre-surgical medication use			
• Non-narcotic analgesics • Anti-inflammatory • Narcotics	18 (41 %) 16 (36 %) 5 (11 %)	25 (24 %) 17 (16 %) 9 (8 %)	*χ* ^2^ = 4.56, *p =* 0.03 *χ* ^2^ = 7.49, *p =* 0.01 FET = 0.30, *p =* 0.58
Type of surgery			
• RC repairs • Resection of clavicle • Acromioplasty • Biceps tenodesis • Biceps tenotomy • Debridement	19 (43 %) 9 (20 %) 41 (93 %) 2 (5 %) 3 (7 %) 13 (30 %)	52 (49 %) 24 (23 %) 96 (91 %) 8 (8 %) 13 (12 %) 40 (38 %)	*χ* ^2^ = 0.83, *p =* 0.36 *χ* ^2^ = 0.09, *p =* 0.77 *χ* ^2^ = 0.27, *p =* 0.60 FET = 0.24, *p =* 0.72 FET = 0.15, *p =* 0.40 *χ* ^2^ = 0.91, *p =* 0.34
Tear size (in cuff repair group)			
• Small • Moderate • Large • Massive	4 (21 %) 11 (58 %) 3 (16 %) 1 (5 %)	6 (12 %) 23 (44 %) 14 (27 %) 9 (17 %)	FET = 0.02, *p =* 0.049

FET Fisher's Exact Test

*χ* ^2^ = Chi square

Wait time1: Symptom duration (months)

Wait time2: Consent date to surgical date (days)

### Mechanism of Injury

In the ESPA group, females had higher incidence of repetitive injuries (*p =* 0.01) with men reporting higher incidence of falls (*p =* 0.01) (Table 1). However, no differences were observed in mechanism of injury in those who were referred for surgical consultation (Table 2) or those who had a rotator cuff related surgery (Table 3).

### Prevalence of pathology

In the ESPA group who had a variety of shoulder pathologies, labral tears occurred exclusively in men and men also had a statistically significant higher prevalence of full-thickness rotator cuff tears (Table 1). Prevalence of rotator cuff impingement syndrome, biceps pathology, adhesive capsulitis, partial-thickness rotator cuff tears, and glenohumeral instability was similar between men and women.

Full-thickness rotator cuff tear was the prominent diagnosis (81/169 = 48 %) of the patients referred for surgery, and although there was a trend towards a higher prevalence of these tears in men (F: 39 % vs. M: 50 %), the difference did not reach statistical significance. Prevalence of all other pathologies was also comparable between men and women referred for surgery (Table 2).

Similarly, in the surgical group who had surgery for rotator cuff pathology, no differences were observed in the frequency of rotator cuff repair, acromioplasty, distal clavicle excision or biceps tenotomy/tenodesis (Table 3). However, among those who had a rotator cuff repair, men had a slightly higher incidence of larger tears than women. Women had a higher rate of small (<1 cm.) (65 % vs. 45 %) and moderate (1–3 cm.) sized tears (18 % vs.10 %) and men had a higher rate of large or massive tears (3 to >5 cm.) (40 % vs.16 %, *p =* 0.049).

### Perceived pain and disability

No statistically significant differences were observed between men and women seen in the ESPA with respect to pain as measured by the NPRS. However, women of the full sample and those who were referred for surgical consultation reported more disability as measured by the QuickDASH (Table 4). In the surgical group, there were no statistically significant differences between men and women with respect to the pre-operative ASES or VAS (Table 4).

**Table 4:** Disparity between men and women in perceived pain and disability

Variables (Min/Max)	Women Mean (SD)	Men Mean (SD)	Statistics *P* values
ESPA group			
NPRS (0/10)	5.9 (2)	5.7(2)	ttest = 1.42, *p =* 0.15
Quick DASH (0/100)	58(22)	54(33)	ttest = 2.27, *p =* 0.02
ESPA subgroup referred for surgical consultation			
NPRS (0/10)	6.24 (2)	5.95(2)	ttest = 0.70, *p =* 0.40
Quick DASH (0/100)	65 (21)	58(19)	ttest = 2.08, *p =* 0.04
Independent Surgical group			
VAS (0/10)	6.7 (2)	6.7(2)	ttest = 0.2, *p =* 0.81
ASES (0/100)	34(16)	33(15)	ttest = 0.39, *p =* 0.70

ASES American Shoulder and Elbow Surgeons, ESPA Early Shoulder Physician Assessment, FET Fisher’s Exact Test, Quick DASH Quick Disabilities of the Arm, Shoulder and Hand, NPRS Numeric Pain Rating Scale

### Medication utilization

In the ESPA group, females took more non-narcotic analgesics, muscle relaxants, and anti-inflammatory medications. Similarly, in the surgical group, females took more non-narcotic analgesics, and anti-inflammatory medications (Tables 1, 2 and 3).

### Gender-related differences

#### Referral pattern

As noted earlier, there was a small effect size difference between men and women of the ESPA group who were referred for assessment. However, a large difference was observed between men and women referred for surgical consultation of whom only 38(22 %) were females with 131(78 %) being males, indicating significant gender disparity. The independent rotator cuff surgical group had a similar discrepancy between women and men (F: 29 vs. M: 71 %) with a medium effect size difference.

#### Wait time

No statistically significant time differences were observed between men and women to see the specialist in the ESPA group (Table 1) or those who were referred for surgical consultation (Table 2). However, the timeframe from injury to surgery (symptom duration) was longer in women who underwent surgery (20 vs. 14 months, *p =* 0.01). A similar gender disparity was observed in the wait-time to have surgery (date patient consented to surgery to the date of actual surgery) with women waiting 95 days vs. men waiting 77 days (*p =* 0.038).

## Discussion

Biological differences in anatomy, strength, hormones, neuromuscular control, and musculoskeletal flexibility can have a negative impact on women’s health. Similarly, gender differences in access to resources, inequalities for being referred for specialist assessment or surgery can have negative health consequences. Although these differences are reported in the general population, there is limited information on sex/gender disparity in injured workers with shoulder pathology. The present study used consecutive injured workers with an active shoulder compensation claim from two different programs designed to expedite assessment and surgical management and showed discrepancy between men and women in terms of mechanism of injury, pathology, medication use, being referred for surgery and waiting to have surgery.

### Mechanism of injury

Previous research has shown that in the general population, men report more traumatic injuries than women, potentially due to their different life style and higher risk-taking behaviors [37–39]. In the present study, where all patients were injured workers, repetitive injuries were more prevalent in women in the ESPA group who were referred for an early assessment. Women more often occupy jobs that involve computer work, prolonged precision demands, awkward postures or repetitive activities [40–42]. In addition, they have less muscle strength and higher reaction time [43] which may explain the higher prevalence of these injuries in women. Men on the other hand had a higher rate of falls on the same level. Of fall injuries treated in Emergency departments, fractures and injuries among older women are reported to be higher than for older men [44]. Unfortunately, the literature on the incidence of falls in working-age women is limited. What is interesting is that despite the fact that women reported more repetitive injuries and men had a higher rate of falls in the ESPA group, these injuries did not differ in the sample of surgical candidates or those who required surgery.

### Prevalence and type of pathology

The present study found that sex of the patient was correlated with the rotator cuff tear size with women having smaller rotator cuff tears than men. In one study [45] that examined 108 women and 171 men, younger women (<55 years of age) had a higher prevalence of small tears compared with their male counterparts. This difference was not statistically significant between older men and women. Considering, the overall women’s smaller stature, older women potentially had more significant tears than men in the same age group. In a systematic review by Oh et al., [12] shoulder pain had a higher prevalence in older female patients of 70 to 79 years of age. An epidemiological study has also reported [46] that rotator cuff pathology is more common in women than in men (90 vs. 83 cases per 100,000 people-years in women and men respectively; *p <* 0.001). The samples used in our study included traumatic rotator cuff tears in the younger working individuals which may explain the higher incidence of pathology in men.

Certain pathologies have a different prevalence in men and women as a result of anatomical differences [47–49] and a higher involvement of men in overhead sports. For example men are approximately three to four times more likely to suffer from neuropathy secondary to suprascapular nerve entrapment syndrome than females [50]. Lack of good epidemiologic studies on labral tears makes the interpretation of prevalence of SLAP tears difficult but some authors [51] have indicated a higher prevalence of this pathology in men, may be due to their involvement in contact and overhead sports activities.

### Sex-related role of hormones on tendon pathology

There is a body of literature on the association between thyroid hormones and tendinopathy [52–58]. Thyroxine has an important role in collagen synthesis and matrix metabolism [59]. Hypothyroidism can cause accumulation of glycosaminoglycans (GAGs) in the extracellular matrix, leading to higher incidence of tendon calcification [58]. In a retrospective study of a large sample of patients (*N =* 441), Oliva et al. [54] reported that thyroid disease was more prevalent in females, independent of age. The prevalence was highest among women in the age group of 60–80 years (women:63 vs. men:23 %) [54] which shows the role of thyroid hormones on modifying and increasing the rate of age related or non-traumatic rotator cuff tear.

Heart et al. [60] have proposed that the higher prevalence of rotator cuff tears in women can be partly explained by hormonal variation in estrogens and thyroxin which may influence collagen and matrix metabolism at a structural and biochemical level [52]. Among co-morbidities, a higher presence of hormone-related gynecologic diseases, autoimmune pathologies, hypothyroidism, rheumatoid arthritis and type 1 diabetes mellitus were found most frequently in women with calcifying tendinopathy [61, 62].

In the present study, the incidence of thyroid condition was not documented and could not be analyzed. It is of note that our sample involved younger women with traumatic pathologies which may reduce the influence of thyroid conditions on development of degenerative rotator cuff pathologies. Sex-related differential role of endocrine disorders that can lead to an early development of shoulder symptoms in women deserves further investigation and should not be underestimated. Future studies should investigate the role of thyroid problems in the development of rotator cuff disease in injured workers.

### Perceived pain and disability

In the present study, a higher disability based on QuickDASH was reported in women in the ESPA group and the ESPA subgroup which is consistent with the available literature. Curry et al. [63] who examined 67 patients with rotator cuff tears undergoing operative and non-operative treatment reported higher disability in women based on the Shoulder Pain and Disability Index (SPADI). Similarly, Harris et al. [64] who reviewed 389 patients with symptomatic atraumatic rotator cuff tears reported the female sex as a negative predictor of ASES scores. Razmjou et al. reported that female candidates for rotator cuff surgery reported higher levels of disability despite similar or lower levels of pathology [39, 45]. Interestingly, in the present study, women who were to undergo surgery did not necessarily perceive themselves as being more disabled than men based on the ASES. This may indicate that female injured workers may learn to adjust or adapt to their functional difficulty as they wait longer to have surgery. However, this discrepancy warrants further investigation.

### Medication use

Women in both groups took more medication. The difference in perception of pain in women has been extensively documented [65–67]. Sex-related neuroanatomical and physiological differences may explain a variety of chronic pain syndromes that are vastly more pervasive in women than men. [68]. Gender related factors such as social conditioning, cultural upbringing, drug dependency traits, negative affect and other psychosocial factors do play a role on pain perception and medication use as well [69]. Acknowledging differences in use of medication in injured workers is critical in providing healthcare services that focus on improving pain and function. This may include facilitating and expediting women’s care (conservative or surgical).

### Referral pattern

The fairly similar percentage of men and women (58 VS. 43 %) seen at the ESPA program reflected by the small effect size suggests a comparable rate of referral of male and female injured workers. According to Stats Canada [70] women in general are more likely to have a regular medical doctor than men (89 % vs. 81 %). In 2009, the largest gender gap in this regard was in the 20-to-34 age group, in which 81 % of women had a regular medical doctor versus only 67 % of men. Partly reflecting the fact that women were more likely to have access to a regular medical doctor, they were also more likely than men to have consulted a doctor in 2009 (86 % vs. 74 %). In Ontario, Canada the access rate was 94 % vs. 89 % in women and men respectively. This may explain why females with injuries did not differ that significantly from their male counterpart in terms of seeing a specialist as even in the presence of potential bias from the family physician or nurse case managers, their tendency to seek more help has neutralized the potential differences.

However, the referral pattern of the surgical candidates of the same sample and the independent sample of rotator cuff pathology group indicates more gender disparity. We observed a large differential pattern of referral (large effect size of 0.55) between men and women who required surgical consultation (ESPA group). This discrepancy was less in magnitude (medium effect size of 0.41) in patients who actually had a rotator cuff-related surgery. In both groups who either needed surgical consultation or underwent surgery, the number of men was significantly higher despite similarity of pathology. Although a higher prevalence of full-thickness rotator cuff and labral tears was observed in men in the ESPA group, this did not explain the discrepancy in the referral pattern of those who needed surgical consultation or underwent surgery. Therefore, the discrepancy in the number of men and women in the surgical groups cannot be explained by higher incidence of pathology and appears to be related to non-biological factors. Our findings are consistent with the literature in general population which indicates that patient’s gender has an impact on physician’s decision to refer a patient for, or to perform certain musculoskeletal surgeries [22–24, 71, 72].

### Wait time differences for surgery

The gender-related difference in wait time to surgery found in this study is consistent with previous studies. Gender disparity in use of health care services has been noted in the literature [73–76]. In addition, women’s persistently social and dominant domestic role in the family dwelling may contribute to postponing their own priorities for the sake of other family members. The gender-specific roles may contribute to longer wait time to surgery [37, 38, 77, 78].

## Limitations

The present study examined basic sex and gender differences in two samples of injured workers seen in a specialized academic institute and was limited to available data. In addition, our results may not be applicable to community-based hospitals where access to specialists is more limited. Although, men and women used the same outcome measures in each sample, there was variability in type of subjective outcomes between two samples. However, these measures have shown high correlation with one another.

Studies that explore work-related gender disparity are influenced by complex political (job availability, pay equality), physical and mental occupational demands, cultural (care giving roles) and social factors (marital status, level of income, access to the health care system, and extent of family and social support). Given the complexity of these relationships, more comprehensive and gender-sensitive measures and analyses are required to capture all important aspects of sex and gender disparity and interaction. More research is needed to examine the differential role of hormones such as thyroxin on tendon pathology and healing.

## Conclusion

Sex and gender disparity exists in workers with shoulder injuries and is evident in the mechanism of injury, perceived disability, medication consumption, referral pattern, and wait time for surgery. Understanding these differences may assist clinicians to customize their management based on the sex of the injured workers. Expedited surgical programs should aim at reducing these differences to assist with lowering the cost of disability in injured workers.

## Abbreviations

ASES: American shoulder and elbow surgeons
ESPA: Early shoulder physician assessment
FET: Fisher’s exact test
MRI: Magnetic resonance imaging
NPRS: Numeric pain rating scale
Quick DASH: Quick disabilities of the arm, shoulder and hand
RTW: Return to work
SD: Standard deviation
US: Ultrasound
